# Outbreak of Parasitic Dinoflagellate *Piscinoodinium* sp. Infection in an Endangered Fish from India: Arulius Barb (*Dawkinsia arulius*)

**DOI:** 10.3390/pathogens11111350

**Published:** 2022-11-14

**Authors:** Arun Sudhagar, Nithianantham Sundar Raj, Sowmya Pazhur Mohandas, Shaji Serin, Konnoth Kuttappan Sibi, Nandiath Karayi Sanil, Thangaraj Raja Swaminathan

**Affiliations:** 1 Peninsular and Marine Fish Genetic Resources Centre, ICAR-National Bureau of Fish Genetic Resources, Ernakulam North P.O., Kochi 682 018, Kerala, India; 2 Fish Health Section, Marine Biotechnology Division, ICAR-Central Marine Fisheries Research Institute, Ernakulam North P.O., Kochi 682 018, Kerala, India

**Keywords:** *Piscinoodinium* sp., parasitic dinoflagellate, indigenous ornamental fish, endangered fish

## Abstract

Freshwater velvet disease is caused by the dinoflagellate parasite, *Piscinoodinium* sp. This parasite has been reported in tropical and subtropical fishes, and it can cause devastating losses. Moreover, *Piscinoodinium* sp. is identified as one of the least studied finfish parasites, and the available molecular information about this parasite is meager. Recently, *Piscinoodinium* sp. was responsible for the 100% cumulative mortality of the captive-bred F1 generation of Arulius barb (*Dawkinsia arulius*), an endangered freshwater fish native to India. The trophont stages of the parasite were observed in the skin and gills of the affected fish. The total DNA was extracted from the trophonts collected from the affected Arulius barb and the partial nucleotide sequence of the rDNA complex region (2334 bp) was amplified using PCR. The amplified PCR product exhibited a high sequence identity (97.61%) with *Piscinoodinium* sp. In the phylogenetic analysis of the SSU rDNA, *Piscinoodinium* sp. emerged as a separate clade from other dinoflagellate species. This is the first report of the infection of *Piscinoodinium* sp. in Arulius barb and the molecular information generated from this study can serve as a baseline to study the diversity of the parasite in India. Furthermore, the impact of this parasite among wild fish stock is not known, and this parasite needs further research focus to generate more molecular information and to understand the host–pathogen interaction.

## 1. Introduction

The parasitic dinoflagellate *Piscinoodonium* sp. is the causative agent of ‘freshwater velvet disease’ or ‘rust disease’, named after the typical clinical sign of a velvety golden dusk-like sheen on the skin of the affected fish [1]. The trophont stages of the parasite are attached to the gills, skin, and fin of the affected fish, which displays a velvet or rust-like appearance. This parasite has a worldwide distribution and a broad host range, particularly in tropical and subtropical fishes [1,2,3,4,5,6]. Furthermore, the parasite has been reported in both ornamental and food fishes [7]. However, very limited molecular information is available about this parasite. In India, a *Piscinoodonium* sp. outbreak was previously reported in common carp (*Cyprinus carpio*), mahseer (*Tor khudree*), and tilapia (*Oreochromis mossambicus*) [8]. The affected fish exhibit extensive epidermal erosion on the skin. The parasite attaches to the host by means of rhizocysts, which cause necrosis, hypertrophy, and lamellar fusion in the gills, subsequently leading to difficulty in respiration [2,9]. Furthermore, the water temperature can play an important role in the outcome of the infection. A previous report suggested that a sudden drop in water temperature can favor *Piscinoodinium* sp. outbreaks in fish [8]. The life cycle of *Piscinoodonium* sp. involves a trophont stage, which attaches to and feeds on the epithelium of the fish host. The trophont detaches from the host and loses its rhizoid-like structure to form tomonts. The tomonts undergo division to form motile dinospores, which can readily infect the fish host and subsequently develop as trophonts, and the cycle continues [10,11]. This life cycle is similar to that of *Amyloodinium* sp., which causes similar disease with the same clinical signs in saltwater fishes. Previously, it was believed that *Piscinoodinium* sp. and *Amyloodinium* sp. were closely related. However, a molecular phylogenetic analysis suggested that these parasitic dinoflagellates are distantly related and underwent convergent evolution to adopt parasitism [12]. Even though *Piscinoodinium* sp. can cause deleterious effects on the affected fish, this parasite has been often overlooked by researchers. Only a few reports of this parasite are available and no detailed studies have been undertaken, particularly on the generation of molecular information about the parasite and to understand the host–pathogen interaction.

The indigenous fish hatchery facility, ICAR—National Bureau of Fish Genetic Resources (NBFGR), Kochi, Kerala, India, serves as a live germplasm-resource centre for threatened endemic fishes, such as Arulius barb (*Dawkinsia arulius*), Narayan barb (*Pethia setnai*), sun catfish (*Horabagrus brachysoma*), Nilgiri mystus (*Hemibagrus punctatus*), and naadan mushi (*Clarias dussumieri*). The indigenous aquarium fish, Arulius barb, which is native to the Tungabhadhra riverine system and upper reaches of the Cauvery basin, was bred in this facility for the purpose of ex situ conservation and river ranching. Moreover, Arulius barb is listed as an endangered species by the International Union for Conservation of Nature (IUCN)’s red list of threatened species [13]. In March 2021, an acute disease outbreak was observed in the hatchery-bred F1 generation of Arulius barb in this facility, eventually leading to complete mortality in the affected stock. A subsequent clinical examination identified *Piscinoodinium* sp. as the causative agent of the outbreak, and this is the first documentation of this parasite in Arulius barb supported by molecular data.

## 2. Materials and Methods

### 2.1. Case History

Breeding of Arulius barb is routinely performed at the indigenous-fish-hatchery facility, ICAR-NBFGR Kochi centre, for conservation. Captive-bred F1-generation Arulius barb (1+ year old) were maintained in 250-L glass tanks at a stocking density of 25 fish per tank. The water-quality parameters were as follows: water temperature, 25 °C to 26 °C; pH, 6.8 to 7.2; dissolved oxygen, 5.5 to 6.0 ppm; ammonia, nil; nitrite, nil; and nitrate <0.01 ppm. The fish were fed thrice a day and the water exchange was performed at a rate of 50% per day. These fishes encountered severe mortality, which eventually led to 100% cumulative mortality (>100 numbers) in 8 days. A total of five moribund fish with a length of 7.2 ± 0.7 cm and a weight of 4.1 ± 0.4 g were examined. The fish were euthanized in 1 g/L of MS222 (Sigma-Aldrich, Saint Louis, MO, USA) before sampling.

### 2.2. Examination of Affected Fish and Collection of Parasites

Necroscopy of the affected fish suggested a severe infestation of the trophonts stages of *Piscinoodinium* sp. in the gills, fins, and body surfaces. The gills along, with the arch, were carefully placed on a Petri plate and observed under a stereomicroscope (Olympus SZ61, Olympus, Tokyo, Japan) to examine the parasite burden. The sizes of the trophont stages of the parasites were measured using CellSens Imaging Software version 1.17 (Evident Corporation, Nagano, Japan). Subsequently, the gills were washed and aspirated with distilled water, and the parasites were carefully collected in a sterile Petri plate using a Pasteur pipette under the stereomicroscope. The trophonts were further washed in two changes of distilled water and collected in a 1.5-mL microcentrifuge tube (Eppendorf, Hamburg, Germany). The trophonts from all five fish were pooled together. The tube was centrifuged at 5000 rpm for 10 min and the supernatant water was discarded. The parasites were then preserved in 100% ethanol and stored at −20 °C for molecular analysis. Samples were also collected from the sampled fish for routine bacterial and viral diagnostics. Briefly, nucleic acid (DNA and RNA) was extracted from kidneys, gills, and brains of the infected animals. DNA was extracted using the salting-out method [14], and RNA was extracted using TRIzol reagent (Invitrogen Carlsbad, USA). The cDNA synthesis was performed from the extracted RNA using the Verso cDNA kit (Thermo Scientific, Vilnius, Lithuania). Subsequently, polymerase chain reaction (PCR) was conducted to check for the presence of viruses, such as cyprinid herpesvirus 2 (CyHV-2) [15], carp edema virus (CEV) [16], infectious-spleen-and-kidney-necrosis virus (ISKNV) [17], and viral nervous necrosis (VNN) [18]. The presence of bacterial infection was examined by aseptically collecting samples from the kidney and spleen of the animals and culturing them in the nutrient medium for bacterial growth.

### 2.3. Polymerase Chain Reaction and Sequencing of rDNA

DNA extraction from the trophonts was performed by the salting-out method [14]. Subsequently, PCR amplification was undertaken by targeting 2334 bp in the rDNA-complex region using GCG18SF and ITSR8 primer pair (Table 1) [12]. The PCR reaction contained 12.5 μL of 2× Emerald Amp GT PCR master mix (Takara, Shiga, Japan), 0.5 μL of each oligonucleotide primer (10 pmol/μL), 10.5 μL of nuclease-free water, and 1 μL containing 50 ng of template DNA. The reaction mixture was initially denatured at 94 °C for 2 min followed by 35 cycles of denaturation (95 °C for 30 s), annealing (58 °C for 45 s), and extension (72 °C for 60 s), and the reaction was then subjected to final extension at 72 °C for 10 min. Subsequently, cloning of the PCR product was performed in pGEM^®^-T easy-vector systems (Promega, Madison, USA) and transformed in the DH5α strain of *Escherichia coli*. The recombinant plasmids were sequenced in ABI Prism 3700 Big Dye sequencer platform (Agrigenome, Kochi, India). The complete sequence of the insert was obtained by primer walking in both 5′ and 3′ directions using the primers provided in Table 1. The obtained sequences were further analyzed in Sequence Scanner Software ver. 1.0 (Applied Biosystems, Foster City, CA, USA) and Bioedit ver. 7.2.5 [19] and assembled into a consensus sequence.

### 2.4. Phylogenetic Analysis

A phylogenetic tree was constructed using the small subunit (SSU) rDNA sequence (1801 bp) of the *Piscinoodinium* sp. from the present study and other representatives (collected from the NCBI database) using MEGA X software [20]. The sequences were aligned using MUSCLE, and the best-fitting model for the construction of the phylogenetic tree was computed to be the Tamura–Nei model with discrete Gamma distribution and invariable sites (T93 + G + I) and a lowest Bayesian Information Criterion score of 15,087.28 and maximum-likelihood value of −6952.84. The maximum-likelihood method was used for constructing the phylogenetic tree with the T93 + G + I model and the test of phylogeny was performed using the Bootstrap method with 1000 replicates. The final tree was visualized with the interactive tree of life (iTOL) [21].

## 3. Results

### 3.1. Clinical Signs and Parasitological Examination

**Figure 1:**
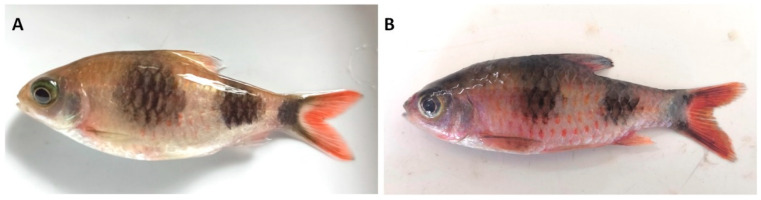
(**A**) Healthy disease-free Arulius barb. (**B**) Arulius barb affected by *Piscinoodinium* sp., showing redness on the body surface.

The affected fish exhibited erratic swimming, anorexia, dyspnea, and excessive mucus production, which eventually led to death. Necroscopy of affected fish showed redness and pinpoint haemorrhage on the body surfaces and fins (Figure 1B) compared to the non-infected fish (Figure 1A). Furthermore, their gills were slightly pale and no gross pathological changes were observed in their other internal organs.

**Figure 2:**
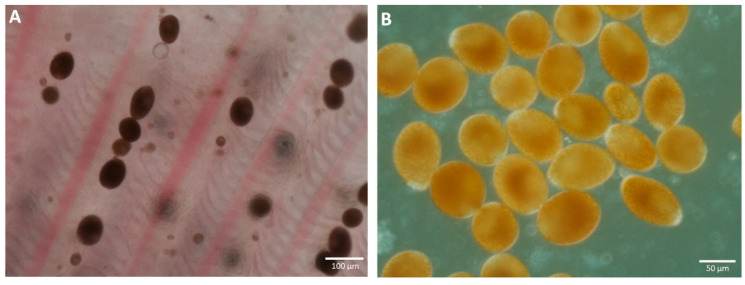
*Piscinoodinium* sp. from Arulius barb visualized under light microscope. (**A**) Trophont attached on the gills. (**B**) Detached trophont collected on a petri plate.

A microscopic observation of the skin-and-gill smear suggested a heavy infestation of the trophonts of the parasitic dinoflagellate *Piscinoodinium* sp. (Figure 2). The highest parasitic burden was observed on the first-gill arch, ranging from 152 to 232 trophonts. However, the other four gill arches had a relatively lower parasite burden with 82 to 110 trophonts. The size of the trophonts was in the range of 15.87 × 19.63 µm to 60.34 × 94.21 µm. In the routine diagnostics using PCR, the animals were observed to be free of CyHV-2, CEV, ISKNV, and VNN viruses and, moreover, no bacterial growth was observed in the nutrient broth inoculated with the kidney and spleen tissues of the infected animals.

### 3.2. Polymerase Chain Reaction and Sequence Analysis

About 2334 bp of rDNA complex region spanning small subunit (SSU) rDNA, internal transcribed spacer 1 (ITS 1), 5.8S rDNA, internal transcribed spacer 2 (ITS 2), and partial sequence of the large subunit (LSU) DNA of *Piscinoodinium* sp. were amplified using PCR (Figure S1). The nucleotide sequence of *Piscinoodinium* sp. generated in this study showed 97.61% similarity to the sequence of *Piscinoodinium* sp. already available in the NCBI GenBank (EF016922.1 and EF016917.1). Furthermore, a search for the nucleotide sequences of *Piscinoodinium* sp. in the NCBI GenBank retrieved only eight sequences from the database. The nucleotide sequence generated from the present study was deposited in the NCBI GenBank under the accession numbers OP452934 (SSU rDNA gene) and OP420760 (ITS region) (Table S1).

### 3.3. Phylogenetic Analysis

**Figure 3:**
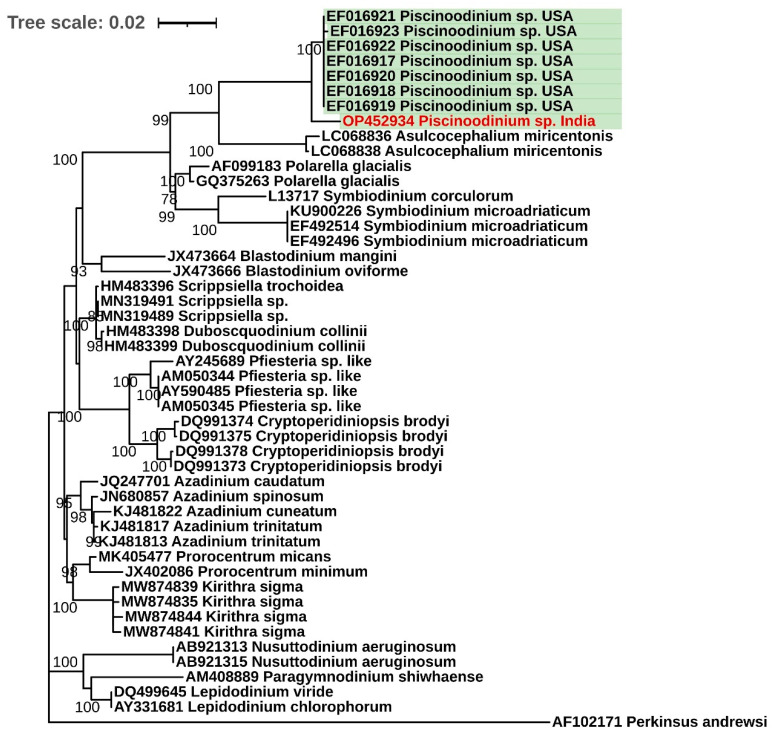
Phylogenetic tree of SSU rDNA gene from selected representatives of dinoflagellates. The maximum likelihood method was used to generate the tree. The green highlights represent the sequences from the parasitic dinoflagellate *Piscinoodinium* sp. and the red font indicates the sequence generated in the present experiment. The SSU rDNA gene sequence from *Perkinsus andrewsi* (accession number AF102171) was used as an outgroup.

The SSU rDNA phylogenetic analysis revealed that the *Piscinoodinium* sp. formed a distinct clade from the other dinoflagellates (Figure 3). The sequence data generated in the present experiment was the first molecular information on *Piscinoodinium* sp. reported from India, and this sequence clustered with seven other sequences of the parasite reported previously from the USA [12]. Moreover, the dinoflagellates belonging to the order Suessiales (*Piscinoodinium* sp., *Asulcocephalium* sp., *Symbiodinium* sp., and *Polarella* sp.) emerged as a separate clade.

## 4. Discussion

Diseases cause morbidity and mortality in fish in aquaculture operations, causing huge production losses in the industry [22,23]. Moreover, diseases in wild fish can lead to population decline, leading to biodiversity losses [24]. It is important to study and understand disease-causing pathogens at the molecular level to develop strategies to prevent outbreaks. The present document is the first report of *Piscinoodonium* sp. in an endangered fish species, Arulius barb. The high parasite burden in the gills of the infected fish might have been the reason for the reduction in respiratory efficiency, which eventually led to 100% cumulative mortality in the Arulius barb. Moreover, we did not find any bacterial or viral co-infection in the infected fish. Previous reports suggested that the *Piscinoodonium* sp. outbreak occurred when the water temperature dropped from 30 °C to 21 °C [8]. In the present study, the water temperature ranged between 25 °C and 26 °C during the disease outbreak. Laboratory experiments suggest that even a slight variation in water temperature can lead to different outcomes of disease during parasitic infection in fish [25,26]. However, detailed experiments are needed to understand the relationship between the drop-in water temperature and *Piscinoodonium* sp. outbreaks in fish. Moreover, studying the host response during *Piscinoodonium* sp. infection can help to understand the immune defence mechanism of the host against the parasite.

Traditionally, dinoflagellates are characterized and identified based on their morphological features. However, with the advent of molecular taxonomy based on conserved nucleic acid sequences, many of the morphology-based classifications of dinoflagellates were found to be inaccurate. This led to the application of the rDNA sequence as a widely used marker to identify and characterize dinoflagellates [27]. In the present study, we targeted an rDNA-complex region (2334 bp) comprising SSU rDNA, ITS 1, 5.8S rDNA, ITS 2, and a partial sequence of LSU DNA of *Piscinoodinium* sp. for PCR amplification. The generated sequence showed a strong homology with the already existing sequences of *Piscinoodinium* sp. reported from the USA [12]. In spite of the various reports of *Piscinoodinium* sp. from the fish hosts, there are no detailed studies aiming to understand the species diversity, molecular taxonomy, and distribution of the parasite, nor on the host–parasite dynamics. Moreover, the molecular information on the *Piscinoodinium* sp. is scarce. Currently, including the present work, there are only eight nucleotide sequences available for *Piscinoodinium* sp. in the NCBI database. Furthermore, there is only one report of *Piscinoodinium* sp. from India [8]. The disease outbreaks due to this parasite in aquaculture operations and the parasite’s prevalence among wild fish populations are poorly documented in India. This overall lack of information about *Piscinoodinium* sp. suggests that the parasite might have been overlooked or poorly studied. There is a need to generate molecular information about this parasite to better understand and develop prophylactic measures against it [28]. Moreover, molecular characterization can help to explore the diversity and evolution of complex parasites [29].

The SSU rDNA gene is identified as one of the most reliable markers with which to determine the phylogenetic relationships among the dinoflagellates. In the phylogenetic analysis of the SSU rDNA gene of *Piscinoodinium* sp. and other representative dinoflagellates, it was observed that the species belonging to the order Suessiales (*Piscinoodinium* sp., *Asulcocephalium* sp., *Symbiodinium* sp., and *Polarella* sp.) emerged as a distinct clade (Figure 3). Among them, only *Piscinoodinium* sp. has a parasitic life cycle with fish as a primary host, and *Symbiodinium* sp. has a symbiotic relationship with a wide range of marine invertebrates. *Asulcocephalium* sp., and *Polarella* sp. are autotrophic dinoflagellates. Moreover, similar to *Piscinoodinium* sp., another parasitic dinoflagellate, *Amyloodinium* sp., causes identical infection and clinical signs in saltwater fishes [1]. However, a phylogenetic analysis suggested that both of these species are genetically distant and might have undergone convergent evolution toward fish ectoparasitism [12].

The affected fish in the present study, Arulius barb, is native to the Cauvery river basin in the Western Ghats region, which is an area of significant biodiversity, harboring diverse fish species [30]. Importantly, Arulius barb is categorized as an endangered species by IUCN [13]. Furthermore, owing to the broad host range and geographical distribution of *Piscinoodinium* sp., this parasite could be a potential ecological threat to the freshwater ichthyofauna in the wild. In Brazil, *Piscinoodinium pillulare* was highly prevalent among the wild population of *Astronotus ocellatus* sampled from a freshwater lake, and seasonality was observed to influence the parasitism [4]. Parasitic infections among wild fish can even lead to the local extinction of the endemic population of the affected fish species [31]. Further research is required to generate knowledge on the species diversity, infection dynamics, geographical distribution, and genomic information of this parasite. Moreover, OMIC technologies can be used to enhance our understanding of *Piscinoodinium* sp. and its host [32].

## 5. Conclusions

This study presents the outbreak of *Piscinoodinium* sp. parasitic infection in a captive-bred endangered fish species, Arulius barb. It is evident that *Piscinoodinium* sp. infection can cause mortality as high as 100% in the affected fish stock. We generated molecular information (rDNA complex region) on *Piscinoodinium* sp., and this information could be used for the molecular diagnosis of the parasite. Furthermore, this sequence information can serve as baseline data for future studies to understand the geographical distribution and diversity of *Piscinoodinium* sp. This parasite deserves research attention in order to prevent devastating outbreaks in aquaculture operations, as well as among wild fish populations.

## Figures and Tables

**Table 1:** List of primers used in this study. The primer pair GCG18SF and ITSR8 was used for the PCR amplification of the rDNA of *Piscinoodinium* sp, whereas primers such as INT_F, INT_R, T7, and SP6 were used for sequencing. The primer sequence of GCG18SF was slightly modified compared to the original reference.

Primer ID	Primer Sequence (5′-3′)	Annealing Temperature (°C)	Target Size (bp)	Purpose	Reference
GCG18SF	CTGGTGATCCTGCCAGTAGTC	58	2334	*Piscinoodinium* sp. PCR	[12]
ITSR8	TAACCTGCATTCATGCTGTG				
INT_F	GTCTGGTGCCAGCAGCCGCGG	58	-	Sequencing	Present study
INT_R	GTACAAAGGGCAGGGACGTA	58	-	Sequencing	Present study
T7	TAATACGACTCACTATAGGG	55	-	Sequencing	Universal primer
SP6	ATTTAGGTGACACTATAG	55	-	Sequencing	Universal primer
CyHV_F	CCCAGCAACATGTGCGACGG	55	362	CyHV-2 PCR	[15]
CyHV_R	CCGTARTGAGAGTTGGCGCA				
CEV_F1	GCTGTTGCAACCATTTGAGA	60	548	CEV nested PCR (outer)	[16]
CEV_R1	TGCAGGTTGCTCCTAATCCT				
CEV_F2	GCTGCTGCACTTTTAGGAGG	55	181	CEV nested PCR (inner)	
CEV_R1	TGCAAGTTATTTCGATGCCA				
ISKNVF	ATGTCTGCAATCTCAGGTGC	55	1362	ISKNV PCR	[17]
ISKNVR	TTACAGGATAGGGAAGCCTG				
VNN_F2	CGTGTCAGTCATGTGTCGCT	55	430	VNN PCR	[18]
VNN_R3	CGAGTCAACACGGGTGAAGA				

## Data Availability

The molecular-sequence data generated in this study were deposited in the NCBI database under the accession numbers OP452934 and OP420760.

## Supplementary Materials

The following supporting information can be downloaded at: https://www.mdpi.com/article/10.3390/pathogens11111350/s1. Figure S1: Detection and amplification of rDNA complex region of *Piscinoodinium* sp. by PCR. Table S1: Nucleotide sequence information of rDNA complex region of *Piscinoodinium* sp. generated in the present experiment.

## References

[1] Noga E.J. (2010). Fish Disease.

[2] Martins M.L., Moraes J.R., Andrade P.M., Schalch S.H., Moraes F.R. (2001). *Piscinoodinium pillulare* (Schäperclaus, 1954) Lom, 1981 (Dinoflagellida) Infection in Cultivated Freshwater Fish from the Northeast Region of Sao Paulo State, Brazil. Parasitological and Pathological Aspects. Braz J. Biol..

[3] Zago A.C., Franceschini L., Garcia F., Schalch S.H.C., Gozi K.S., Silva R.J. (2014). da Ectoparasites of Nile Tilapia (*Oreochromis Niloticus*) in Cage Farming in a Hydroelectric Reservoir in Brazil. Rev. Bras. Parasitol. Vet..

[4] Neves L.R., Pereira F.B., Tavares-Dias M., Luque J.L. (2013). Seasonal Influence on the Parasite Fauna of a Wild Population of *Astronotus ocellatus* (Perciformes: Cichlidae) from the Brazilian Amazon. J. Parasitol..

[5] Shaharom-Harrison F.M., Anderson I.G., Siti A.Z., Shazili N.A.M., Ang K.J., Azmi T.I. (1990). Epizootics of Malaysian Cultured Freshwater Pond Fishes by *Piscinoodinium pillulare* (Schaperclaus 1954) Lom 1981. Aquaculture.

[6] Wilson J.R., Saunders R.J., Hutson K.S. (2019). Parasites of the Invasive Tilapia *Oreochromis mossambicus*: Evidence for Co-Introduction. Aquat. Invasions.

[7] Noga E.J., Levy M.G. (2006). Phylum Dinoflagellata. Fish Diseases and Disorders. Volume 1: Protozoan and Metazoan Infections.

[8] Ramesh K.S., Mohan C.V., Shankar K.M., Ahmed I. (2002). *Piscinoodinium* Sp. Infection in Juveniles of Common Carp (*Cyprinus Carpio*), Mahseer (*Tor Khudree*) and Tilapia (*Oreochromis mossambicus*). J. Aquac. Trop..

[9] Ferraz E., Sommerville C. (1998). Pathology of *Piscinoodinium* Sp. (Protozoa: Dinoflagellida), Parasites of the Ornamental Freshwater Catfishes *Corydoras* spp. and *Brochis splendens* (Pisces: Callichthyidae). Dis. Aquat. Organ..

[10] van Duijn C. (1973). Diseases of Fish.

[11] Jacobs D.L. (1946). A New Parasitic Dinoflagellate from Fresh-Water Fish. Trans. Am. Microsc. Soc..

[12] Levy M.G., Litaker R.W., Goldstein R.J., Dykstra M.J., Vandersea M.W., Noga E.J. (2007). *Piscinoodinium*, a Fish-Ectoparasitic Dinoflagellate, Is a Member of the Class Dinophyceae, Subclass Gymnodiniphycidae: Convergent Evolution with *Amyloodinium*. J. Parasitol..

[13] Abraham R. *Dawkinsia Arulius*; The IUCN Red List of Threatened Species 2015: e.T172500A70168511. https://www.iucnredlist.org/species/172500/70168511.

[14] Miller S.A., Dykes D.D., Polesky H.F. (1988). A Simple Salting out Procedure for Extracting DNA from Human Nucleated Cells. Nucleic Acids Res..

[15] Jeffery K.R., Bateman K., Bayley A., Feist S.W., Hulland J., Longshaw C., Stone D., Woolford G., Way K. (2007). Isolation of a Cyprinid Herpesvirus 2 from Goldfish, *Carassius auratus* (L.), in the UK. J. Fish. Dis..

[16] Oyamatsu T., Matoyama H., Yamamoto K.-Y., Fukuda H. (1997). A Trial for the Detection of Carp Edema Virus by Using Polymerase Chain Reaction. Aquac. Sci..

[17] Swaminathan T.R., Raj N.S., Preena P.G., Pradhan P.K., Sood N., Kumar R.G., Sudhagar A., Sood N.K. (2021). Infectious Spleen and Kidney Necrosis Virus-associated Large-scale Mortality in Farmed Giant Gourami, *Osphronemus Goramy,* in India. J. Fish. Dis..

[18] Nishizawa T., Mori I.K., Nakai T., Furusawa I., Muroga K. (1994). Polymerase Chain Reaction (PCR) Amplification of RNA of Striped Jack Nervous Necrosis Virus (SJNNV). Dis. Aquat. Organ..

[19] Hall T. (1999). BioEdit: A User-Friendly Biological Sequence Alignment Editor and Analysis Program for Windows 95/98/NT. Nucleic Acids Symp. Ser..

[20] Kumar S., Stecher G., Li M., Knyaz C., Tamura K. (2018). MEGA X: Molecular Evolutionary Genetics Analysis across Computing Platforms. Mol. Biol. Evol..

[21] Letunic I., Bork P. (2021). Interactive Tree Of Life (ITOL) v5: An Online Tool for Phylogenetic Tree Display and Annotation. Nucleic Acids Res..

[22] Murray A.G., Peeler E.J. (2005). A Framework for Understanding the Potential for Emerging Diseases in Aquaculture. Prev. Vet. Med..

[23] Lafferty K.D., Harvell C.D., Conrad J.M., Friedman C.S., Kent M.L., Kuris A.M., Powell E.N., Rondeau D., Saksida S.M. (2015). Infectious Diseases Affect Marine Fisheries and Aquaculture Economics. Ann. Rev. Mar. Sci..

[24] Russell R.E., DiRenzo G.V., Szymanski J.A., Alger K.E., Grant E.H.C. (2020). Principles and Mechanisms of Wildlife Population Persistence in the Face of Disease. Front. Ecol. Evol..

[25] Bailey C., Segner H., Casanova-Nakayama A., Wahli T. (2017). Who Needs the Hotspot? The Effect of Temperature on the Fish Host Immune Response to *Tetracapsuloides bryosalmonae* the Causative Agent of Proliferative Kidney Disease. Fish Shellfish. Immunol..

[26] Bailey C., Schmidt-Posthaus H., Segner H., Wahli T., Strepparava N. (2018). Are Brown Trout *Salmo trutta fario* and Rainbow Trout *Oncorhynchus mykiss* Two of a Kind? A Comparative Study of Salmonids to Temperature-Influenced *Tetracapsuloides bryosalmonae* Infection. J. Fish. Dis..

[27] Ott B.M., Litaker R.W., Holland W.C., Delwiche C.F. (2022). Using RDNA Sequences to Define Dinoflagellate Species. PLoS ONE.

[28] Shivam S., El-Matbouli M., Kumar G. (2021). Development of Fish Parasite Vaccines in the OMICs Era: Progress and Opportunities. Vaccines.

[29] Fariya N., Kaur H., Singh M., Abidi R., El-Matbouli M., Kumar G. (2022). Morphological and Molecular Characterization of a New Myxozoan, *Myxobolus grassi* sp. Nov. (Myxosporea), Infecting the Grass Carp, *Ctenopharyngodon idella* in the Gomti River, India. Pathogens.

[30] Sreenivasan N., Mahesh N., Raghavan R. (2021). Freshwater Fishes of Cauvery Wildlife Sanctuary, Western Ghats of Karnataka, India. J. Threat. Taxa.

[31] Sudhagar A., Kumar G., El-Matbouli M. (2019). The Malacosporean Myxozoan Parasite *Tetracapsuloides bryosalmonae*: A Threat to Wild Salmonids. Pathogens.

[32] Sudhagar A., Kumar G., El-Matbouli M. (2018). Transcriptome Analysis Based on RNA-Seq in Understanding Pathogenic Mechanisms of Diseases and the Immune System of Fish: A Comprehensive Review. Int. J. Mol. Sci..

