# Supplementary material for: Outbreak of Parasitic Dinoflagellate Piscinoodinium sp. Infection in an Endangered Fish from India: Arulius Barb (Dawkinsia arulius)
Source: Pathogens. 2022 Nov 14;11(11):1350. doi: 10.3390/pathogens11111350 (PMC9695384; doi:10.3390/pathogens11111350)
Supplement: Supplementary file 1 [file pathogens-11-01350-s001.zip › Figure S1.pdf]

Figure S1: Detection and amplification of rDNA complex region of *Piscinoodinium sp.* by PCR

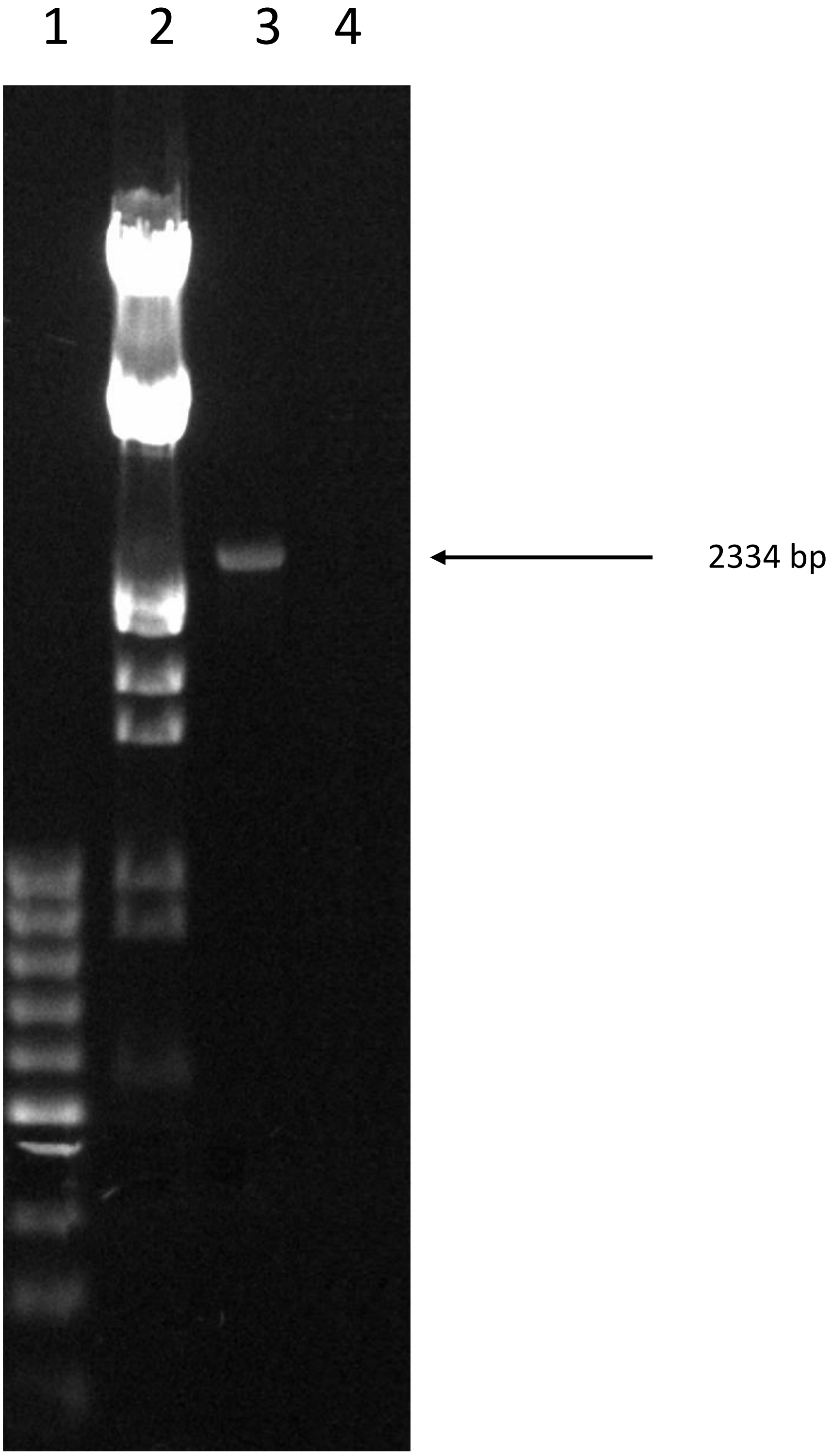

Lane 1 – 100 bp marker
Lane 2 – Lambda DNA/EcoR I/Hind III Double Digest marker
Lane 3 - *Piscinoodinium sp.* collected from the gill of Arulius barb
Lane 4 – Non-template control
