# Supplementary material for: Outbreak of Parasitic Dinoflagellate Piscinoodinium sp. Infection in an Endangered Fish from India: Arulius Barb (Dawkinsia arulius)
Source: Pathogens. 2022 Nov 14;11(11):1350. doi: 10.3390/pathogens11111350 (PMC9695384; doi:10.3390/pathogens11111350)
Supplement: Supplementary file 1 [file pathogens-11-01350-s001.zip › Table S1.pdf]

Table S1: Nucleotide sequence information of rDNA complex region of *Piscinoodinium* sp. generated in the present experiment.

>Present experiment

CTGGTGATCCTGCCAGTAGTCATATGCTTGTCTCAAAGATTAAGCCATGCATGTCTCAGCATAAGCCTTCATAT
GGTGAAGCTGCGAATGGCTCATTAAAGCAGTTATAATTTATTTGATGGTCACTGTTACATGGATAACTGTGGT
AATTCTAGAGCTAATACATGCATCAAAACCCGACCTCTTGGAAGGGTTGTGTTTATTAGGTACAAAACCAACCC
AAGTTCGCTTGGTTCTCTGGTGATTTATGATAACTGAATGAATTGTATGGCATTAGCCGATGATACTTCATTCA
AGTTTCTGACCTATCAGCTTCCGACGGTAGGGTATTGGCCTACCGTGGCAATGACGGGTAACGGAGAATTAG
GGTTTGATTCCGGAGAGGGAGCCTGAGAAACGGCTACCACATCTAAGGAAGGCAGCAGGCGCGCAAATTAC
CCAATCCTGACATAGGGAGGTAGTGACAAGAAATAACAATACAGGGCATCCATGTCTTGTAATTGGAATGAA
TAGAATTTAAATCCCTTTATGAGTATCAATTGGAGGGCAAGTCTGGTGCCAGCAGCCGCGGTAATTCCAGCTC
CAATAGCGTATATTAAAGTTGTTGCGGTTAAAAAGCTCGTAGTTGGACTTCTGCTGAAAAATGACTGGTCCGC
CCTCTGAGCAGGTATCAGGTTCAGCTTTGGCATCTTCTTAAAAGAGCGCGTCTGCACTTTGATTGTGTGGTGCG
GTATTTAAGACATTTACTTTGAAGAAATTAGAGTGTTTCAAGCAAGCGCACGCTTTGAATACATTAGCATGGA
ATAATAAGATAGGACCTCAATTCTATTTTGTTGGTTTCTAGAACTGAGGTAATGATTGATAGGGATAGTTGGG
GGCATTCGTATTTAATTGTCAGAGGTGAAATTCTTGGATTTATTAAAGACGGACTACTGCGAAAGCATTTGCCA
AGGATGTTTTCATTGACCAAGAACGAAAGTTAGGGGATCGAAGACGATCAGATACCGTCCTAGTCTTAACCAT
AAACTATGCCGACTAGAGATTGAAGGTCGTTACTTGTACGACTCCTTCAGCACCTTATGAGAAATCAAAGTCTT
TAGGTTCCGGGGGGAGTATGGTCGCAAGGCTGAAACTTAAAGGAATTGACGGAAGGGCACCACCAGGAGTG
GAGCCTGCGGCTTAATTTGACTCAACACGGGGAAACTTACCAGGTCCAGACATAGTAGGGATTGACAGATTG
AAAGCTCTTTCTTGATTCTATGGGTGGTGGTGCATGGCCGTTCTTAGTTGGTGGAGTGATTTGTCTGGTTAATT
CCGTTAACGAACGAGACCTTAACCTGCTAAATAGTTACATGTAATTTCGGTTACATGGGCAGCTCCTTAGAGG
GACTTTGTGTGTCTAATGCAAGGAAGTTTGAGGCAATAACAGGTCTGTGATGCCCTTAGATGTCCTGGGCTGC
ACGCGCGCTACACTGATGCATTCAACGAGTTATGACCTTGCCTGAAAGGGTTGGGTAATCTTTTTAAATTGCAT
CGTGATGGGGATAGATCATTGCAATTATTGATCTTCAACGAGGAATTCCTAGTAAGCGCGAGTCATCAGCTCG
TGCTGATTACGTCCCTGCCCTTTGTACAAGCCGCCCGTCGCTCCTACCGATTGAGTGATCCGGTGAATAATTCA
GACTGATTCAGTGCTCAGCTTCTGGATACTGCGTCGGAAAGTCTAGTGAACCTTATCACTTAGAGGAAGGAGA
AGTCGTAACAAGGTTTCCGTAGGTGAACCTGCGGAAGGATCATTCGCACAATCCATTCAACTCACTGTGAATG
TAACTGGTAAATGGATGTGGGTAGATGGATGTTTTTGAAACACTTCCTGCTTGCAACCATTTGCGGGTGGCAA
AGCAAACTGCTGCGTGCTGGGCGGTTTTGTTCCTGCTGCCATTAGATTTGTTTTTGCAATCTAATTAATCTAAGT
TTTGAAGCAGTATCTAAACAAATTACAACTTTCAGCGATGGATATCTCGGCTCAAACATCGATAAAGGGCGCA
GCAAAGTGTGATAATCATTGTGAATTGCAGAATTCCGTGAACCAATTGACTTTTGAATGTATATTACACTTCTG
GGTTATCCCTGGATGTTCATCTGCTTCAGTGCTTTGTGTTTCGAATAATTTCATGAATTGTGTTTGTGAGCTATC
AGAAGCATACTAGCTTGTGGTGGCACATCAAAGATAGTTGGTTCATCAGCAACGTTTGTTGCAAGTGGTTAGC
AATAAGGCATCAACTTGCAATAGGCTGCTGGGACAAAACAATATCACAGCATGAATGCAGGTTA

Nucleotide position	Name of the gene	Remarks	Accession number
1 – 1801 bp	Small subunit ribosomal RNA gene	SSU rRNA gene	OP452934
1802 – 2000 bp	Internal transcribed spacer 1	ITS region	OP420760
2001 – 2162 bp	5.8S ribosomal RNA		
2163 – 2314 bp	Internal transcribed spacer 2		
2315 – 2334 bp	Large subunit ribosomal RNA	LSU rRNA gene	Not submitted
